# Isolation and Cytotoxic Investigation of Flacourtin from *Oncoba spinosa*

**DOI:** 10.3390/medicines3040031

**Published:** 2016-12-06

**Authors:** Olaoye S. Balogun, Olukayode S. Ajayi, Olayinka S. Lawal

**Affiliations:** Department of Chemistry, Obafemi Awolowo University, Ile-Ife 220005, Nigeria; osajayi@oauife.edu.ng (O.S.A.); lawalolayinka@gmail.com (O.S.L.)

**Keywords:** *Oncoba spinosa*, Flacourtin, cytotoxicity, Flacourtiaceae, phenolic ester glycoside

## Abstract

**Background:** *Oncoba spinosa,* an endangered medicinal plant whose secondary metabolites have not been extensively profiled, and which is hitherto yet to be examined for cytotoxicity, is being investigated in this study. **Methods:** Leaves of *Oncoba spinosa* (800 g) were extracted with 95% aqueous methanol. The crude extract was partitioned with n-hexane and the resultant defatted extract was extensively chromatographed on silica gel to yield compound **1** which was subjected to spectroscopic analysis. A brine shrimps lethality test was used to establish the cytotoxicity potentials of the isolated compound and the plant extracts. **Results:** Compound **1** was elucidated as flacourtin, 3-hydroxy-4-hydroxymethylphenyl-6-*O*-benzoyl-β-d-glucopyranoside. The LD_50_ values obtained were less than 1000 µg/mL for flacourtin and the plant extracts. **Conclusion:** Flacourtin is being reported for the first time in the *O. spinosa*. The preliminary toxicity assay indicated that flacourtin and the plant extracts were not cytotoxic; thus, the tradomedicinal uses of the plant may portend no danger.

## 1. Introduction

*O. spinosa* is a spiny shrub used for the management of arthritis in southwest Nigeria [1]. The fruit is used for the treatment of anthelminthic, syphilis, wound and sexual impotence [2]. In profiling the chemical constituent of *Oncoba spinosa*, (Flacourtiaceae) five flavonoids, kaempferol, quercetin, apigenin-7-*O*-β-d-glucuronopyranoside, quercetin 3-*O*-β-d-galactopyranoside and quercetin 3-*O*-α-rhamnopyranosyl (1→6) β-d-glucopyranoside have been reported [3]. Flacourtin, a phenolic glycoside ester (3-hydroxy-4-hydroxymethylphenyl-6-*O*-benzoyl-β-d-glucopyranoside) which has been associated with a number of pharmacological activities was first isolated from the bark of a medicinal plant, *Flacourtia indica* (Flacourtiaceae) [4,5,6,7,8], an indigenous medicinal plant widely distributed in India and Bangladesh [9]. Also, Amarasinghe et al. [10] reported isolation of a new glucoside, flacourside, an analog of flacourtin from *Flacourtia indica*. As part of our on-going work on metabolite profiling of indigenous medicinal plants, leaves of *O. spinosa* were collected with the aim of isolating a bioactive compound new to the plant specie.

## 2. Materials and Methods

### 2.1. Plant Collection and Purification

Fresh leaves *Oncoba spinosa* were collected at the Botanical Garden University of Ibadan, Oyo State, Nigeria in the month of November, 2014. The plant was identified by the taxonomist and curator, Mr A. Owolabi in the Botanical Garden. A voucher copy of the plant with herbarium number FHI 108806 was deposited at the Forestry Research Institute of Nigeria, Ibadan.

The air-dried and pulverized leaves (800 g) were extracted with 95% aqueous methanol to give a crude extract (140 g). The crude was reconstituted in methanol and defatted with n-hexane to afford 80.60 g of defatted extract. Twenty grams of the defatted extract was chromatographed on silica gel using gradient of dichloromethane and methanol to give a sub-fraction (1.22 g) which was further purified on silica with gradient of hexane, ethyl acetate and methanol. Compound **1** (6 mg) was obtained as an off-white powder which was recrystallized in ethylacetate and thereafter subjected to spectroscopic analysis.

### 2.2. Cytotoxicity Assay

Cytotoxicity of the crude extract, defatted fraction and compound **1** were investigated using brine shrimp lethality assay. A spoonful quantity of the egg of brine shrimps (*Artemia salina* Leach) was sprinkled into a partly covered crucible containing seawater in order to allow partial illumination. The eggs hatched into matured *nauplii* after about 48 h and 10 matured *nauplii* were added to varying concentration of the test samples (1000, 100 and 10 µg/mL). All the experiments were carried out in triplicate and distilled water was used in place of the test samples for negative control. Numbers of survivors were counted after 24 h and the LC_50_ was computed at 95% confidence limit using the Finney program. 

## 3. Results 

**IR ν_max_ (cm^−1^):** 3000 (ArO–H str), 3050 (Ar–H str), 1710 (C=O str), 1600 (Ar C–C str), 1220 (C–O ester), 1053 (C–O alc).

**^13^C-NMR (MeOD, 75 MHz):** δc 134.5 (C_-1′_), 130.7 (C_-2′_ and C_-6′_), 129.7 (C_-3′_ and C_-5′_), 132 (C_-5′_), 150.1 (C_-1″_), 116.3 (C_-2″_), 154 (C_-3″_), 134.1 (C_-4″_), 115.7 (C_-5″_), 119.7 (C_-6″_), 61.1 (C-_7″_), 104.6 (C_-1_), 75.7 (C_-2_), 75.2 (C_-3_), 72.1(C_-4_), 78.1 (C_-5_), 64.8 (C_-6_), 182 (C=O).

**^1^H-NMR (MeOD, 300 MHz):** δ 8.05 (2H, m, *J_o_* 7.2 Hz, *J_m_* 1.5 Hz, H_-2_ and H_-6_), 7.53 (2H, m, *J*_3,2_ 7.2 Hz, *J*_3,5_ 7.8 Hz , H_-3_ and H_-5_), 7.66 (1H, m, *J_o_* 7.5 Hz, *J_m_* 1.5 Hz, H_-5_), 6.77 (1H, d, *J_m_* 3.0 Hz, H_-2″_), 7.03 (1H, d, *J_o_* 8.7 Hz, H_-5″_), 6.48 (1H, dd, *J_o_* 8.7 Hz, *J_m_* 3.3 Hz, H_-6″_), 4.62 (1H, d, H_-1_), 3.51 (3H, m, H_-2_, H_-3_ and H_-4_), 3.73 (1H, m, *J*_5,6_ 7.5 Hz, H_-5_), 4.46 (1H, d, *J*_5,6_ 7.5 Hz, H_-6a_), 4.42 (IH, d, *J*_5,6_ 7.5 Hz, H_-6b_), 4.72 (H, H_-6a″_), 4.76 (1H, H_-6b″_), 4.95, 4.82 and 4.72 (glucose OH), 4.69 (benzylic OH).

## 4. Discussion

**Figure 1:**
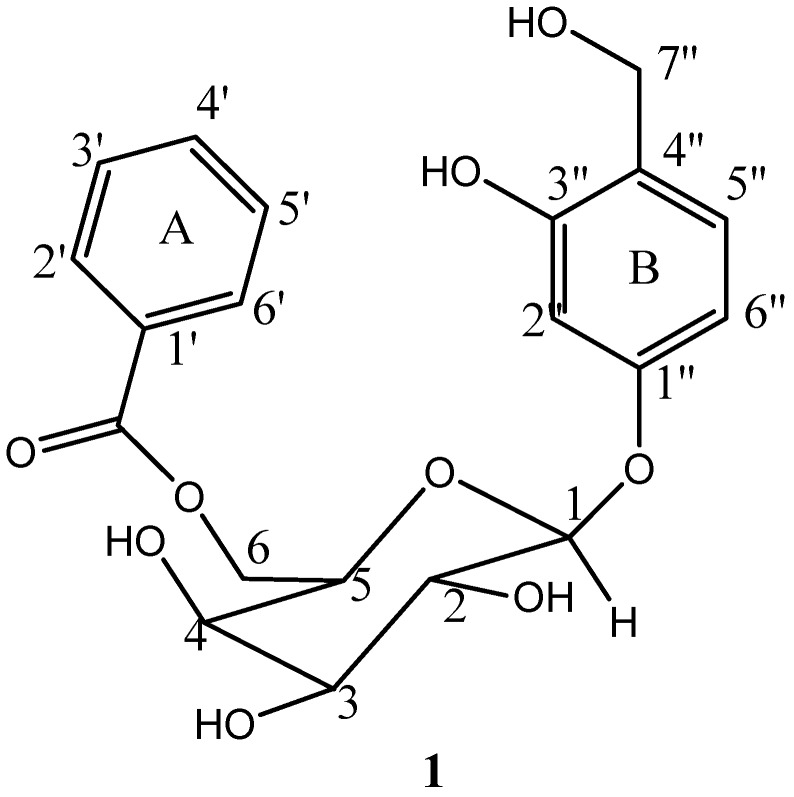
Flacourtin.

The NMR spectra of compound **1** showed a pattern characteristic of a phenolic ester glycoside. Clusters of peaks at δ 3.5–5.0 indicated the presence of a sugar moiety while the aromatic region showed the presence of eight protons with *J* values typical of an aromatic system, thus suggesting two substituted benzene rings (Figure 1). The IR spectrum showed absorption bands (cm^−1^) at 3000 (ArO–H str), 3050 (Ar–H str), 1710 (C=O str), 1600 (Ar C–C str), 1220 (C–O ester), 1053 (C–O alc) and 810 (1,2,4-trisubstituted aromatic). The chemical shift δ 61.1–78.1 ppm in the ^13^C-NMR spectrum indicated *O*-linked carbons of the sugar moiety and the carbinol carbon of the phenolic aglycone. The anomeric carbon and the carbonyl of ester resonated at δ 104.6 and 182 ppm respectively while the aromatic carbons were observed at δ 115.7–150.1 ppm. The spectrum of ^1^H NMR indicated a multiplet at δ 3.50 assignable to three methine protons on the glucose moiety and two doublets of carbinol protons at δ 4.46 (1H, d, *J*_5,6_ 7.5 Hz) and δ 4.42 (IH, d, *J*_5,6_ 7.5 Hz) coupling with H_-5_ at δ 3.73 ppm (1H, m, *J*_5,6_ 7.5 Hz). The anomeric proton appeared as a doublet at δ 4.62 ppm indicating an axial position to the pyranose ring. 

Three aromatic protons of ring B with *J* values attributable to a 1,2,4-trisubstituted aromatic system showed at δ 7.03, 6.77 and 6.48. The two protons each at ortho and meta position in ring A are in the same chemical environment, thus both protons at ortho position resonated at δ 8.05 with *J_o_* and *J_m_* of 7.2 and 1.5 Hz respectively. Similarly, symmetrical protons at meta positions were observed at δ 7.53 with coupling constant of 7.2 and 7.8 Hz, showing that the protons were doubly ortho-coupled, which connoted that ring A is monosubstituted. All the spectroscopic data and their assignments were in good agreement with the report of Bhaumik et al. [4]. The compound (Figure 1), which was isolated for the first time from leaves of *O. spinosa*, was elucidated as 3-hydroxy-4-hydroxymethylphenyl-6-*O*-benzoyl-β-d-glucopyranoside, previously named flacourtin.

The brine shrimps lethality test indicated that crude extract, methanol fraction and flacourtin had LD_50_ values greater than 1000 µg/mL (Table 1) which implied that they exhibited no cytotoxic activity on the *nauplii*.

## 5. Conclusions 

Flacourtin is being reported for the first time in the *O. spinosa*. The preliminary toxicity profile as indicated by brine shrimp lethality test showed that the compound and the plant extracts were not cytotoxic; therefore, the tradomedicinal uses of the plant may portend no danger.

## Figures and Tables

**Table 1:** Cytotoxicity of test samples.

Sample	Crude	n-Hexane Fraction	Defatted Fraction	Flacourtin
**LD_50_ (µg/mL)**	1203	>10,000	>10,000	>10,000
